# The Chase epicardial stabiliser as an adjunct tool for the management of penetrating cardiac injuries

**DOI:** 10.1308/003588412X13373405387050a

**Published:** 2012-10

**Authors:** T Vu, FN Mazzini, JA Asensio

**Affiliations:** University of Miami, FL,US

## BACKGROUND

Penetrating cardiac injuries pose difficult and technical challenges for both trauma surgeons and trauma centres as well as incurring high mortality.^1–3^

## TECHNIQUE

For patients arriving in cardiopulmonary arrest, emergency department thoracotomy is indicated. For those patients arriving stable enough to be transported to the operating room, median sternotomy is the incision of choice. Penetrating injuries to the heart will inevitably cause bleeding of the myocardium, mandating repair to prevent cardiac tamponade. Suture repair of a cardiac laceration is usually carried out with simple interrupted or horizontal mattress sutures but this is quite challenging in beating hearts. At times, rapid manipulation and elevation of the heart during repair results in complex dysrhythmias and even cardiac arrest.^4^

**Figure 1:**
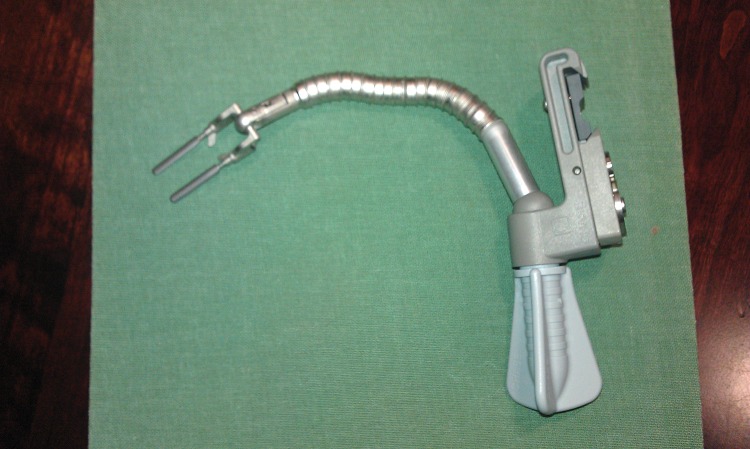
epRetract™ II epicardial retracting stabiliser

In selected cases where the cardiac injury is located in areas difficult to repair (eg in the lateral aspects of the ventricles and those adjacent to the left anterior descending coronary artery), the use of a cardiac stabilisation device with adjustable footplates such as the epRetract™ II epicardial retracting stabiliser (Chase Medical, Richardson, TX, US; Fig 1) may stabilise the heart to allow controlled repair of the lacerated myocardium. This device could be attached easily to the sternum retractor and then the footplates may be positioned parallel or at a 90º angle to the cardiac injury. The footplates may be adjusted to approximate the wound edges and stabilise the heart to facilitate repair.

## DISCUSSION

We recommend the selective consideration and use of this device as an adjunct for suture repair in cases where minimising cardiac manipulation is advantageous.
